# Controlling Sleeping Sickness—“When Will They Ever Learn?”

**DOI:** 10.1371/journal.pntd.0000609

**Published:** 2010-05-25

**Authors:** David Molyneux, Joseph Ndung'u, Ian Maudlin

**Affiliations:** 1 Centre for Neglected Tropical Diseases, Liverpool School of Tropical Medicine, Liverpool, United Kingdom; 2 FIND Diagnostics, Geneva, Switzerland; 3 Centre for Infectious Diseases, College of Medicine and Veterinary Medicine, The University of Edinburgh, Summerhall, Edinburgh, United Kingdom; Yale School of Public Health, United States of America

The recent announcement that WHO has approved the use of a combination of nifurtimox and eflornithine to treat chronic Gambian sleeping sickness, caused by *Trypanosoma brucei gambiense*, is a welcome step in the seemingly interminable process of searching for less toxic drugs to treat this devastating disease [1]. Arsenical drugs were first used in 1905; melarsoprol remains the drug most frequently used for late stage disease and is a drug for which resistance is now a major problem [2].

Over the last 50 years the needs of countries afflicted by sleeping sickness and of the foci of infection have changed little, and neither have our priority needs for research and disease management: cheap point-of-care diagnostics and effective, non-toxic, and affordable drugs for late stage, or stage 2 disease. What is standing in the way of attaining these apparently modest research aims? Surprisingly, one problem is the very nature of the trypanosome and its vector the tsetse fly; these beautiful and biologically fascinating creatures continue to attract considerable research funding, resulting in a burgeoning industry; a PubMed search for *Trypanosoma brucei* reveals 2,624 papers published in the last decade producing outputs that, while admittedly elegant, are remote from the needs of patients from afflicted rural populations and are disproportionate to the sums needed to support research to assist disease management. Could it be that, as development economists suspect, “we have here a silent conspiracy of professional interests whose scientific work is justified on the basis of poverty reduction but who would be devastated if they were actually successful in these terms?” [3]. It would be timely now to take a very hard look at the global research agenda within the context of a forgotten hinterland which, up until the 1960s, demonstrated that this disease could be controlled effectively by unsophisticated means—a history all too conveniently forgotten by, or perhaps unknown to, most of the current generation of researchers.

The ability of the medical services to translate effective tools and technologies into public health successes when faced with the devastating epidemics of the past was dependent on dedicated teams, skilled staff, and adequate and appropriate financing. In West Africa, epidemics of Gambian sleeping sickness were controlled by the use of chemoprophylactic treatment or “pentamidisation” of populations led by Jamot and military style campaigns; in East and southern Africa where the authorities were equally concerned with the health of livestock, the diagnosis and treatment approach for Rhodesian sleeping sickness was allied to vector control [4]. Targeted, effective, and appropriate research (supported largely by French and British aid) allied to realistic health service delivery options worked, and by the 1960s sleeping sickness was not considered a significant public health problem. The numbers of new cases each year was minimal and controlled effectively in all endemic countries of West and Central Africa through active screening by mobile teams who diagnosed cases by microscopy (gland puncture and lumbar puncture) and treated patients with pentamidine or suramin and melarsoprol as appropriate. Whilst there were relapses, there was also regular follow-up and the observed trend towards increasingly frequent detection of early disease was a testament to the effectiveness of the system. For *T. b. gambiense*, diagnosis was improved initially by the use of immunofluorescence tests and later, the more practical card agglutination test for trypanosomiasis (CATT) developed in the 1970s. The CATT test is perhaps the sole relevant product deployed at any scale to emerge from the huge amount of research resources devoted to trypanosome antigenic variation. Yet today, the CATT test remains largely underused, due to the cost of the product and packaging (in units of 50), working out at around US$2 per test [5].

The launch of the Drugs for Neglected Diseases Initiative (DNDi) has focused attention on the need for new drugs for sleeping sickness as well as other kinetoplastid infections (*T. cruzi* and *Leishmania*). The registration of the nifurtimox/eflornithine combination marks progress in improvement of the treatment option for patients with *T. b. gambiense*—albeit at a snail's pace, given that van Nieuwenhoeve did the initial work on eflornithine in 1985 (he also had the vision to suggest the use of nifurtimox for relapse cases) [6]. Seventeen years later, adoption of even a small improvement in treatment regimes is a step forward. As the trypanosome biochemist Jim Williamson so cogently remarked “there have been many more reviews of trypanosome chemotherapy than new drugs” [7]. However, the challenge of the eflornithine/nifurtimox option, even if this combination therapy is available as an “essential drug,” is classic: transport of a weighty product; the difficulties of intravenous administration in rural settings where health facilities are minimal; drug availability and affordability; the intensity of the specialised medical care required for patients; the monitoring of side effects and the potential for relapses requiring regular follow-up: all costly activities where patients are beyond the end of the road. WHO has reported a significant decline in the numbers of new cases over the last five years, indicating that sleeping sickness is coming under control [8], but we must add a proviso: data on sleeping sickness deaths are subject to gross errors due to under-reporting [9] as the majority of people affected are beyond the reach of health care systems and are not reported in any of the health metrics [10]. However, any apparent improvement in incidence is the result of the deployment of classical approaches as opposed to any new advance in therapy. The agreement by Sanofi-Aventis and Bayer to donate the necessary drugs to WHO to distribute to affected countries has been critical; without this generosity, patients would have no access to life-saving drugs, however unsatisfactory, when national health budgets are so stretched.

The tsetse fly, like the trypanosome, also fascinates scientists but we should be aware that tsetse can be eliminated or their populations dramatically reduced by the simplest of technologies. Sleeping sickness was eliminated from the island of Principe in the early years of the 19^th^ century by use of sticky backpacks that trapped tsetse. Morris reduced the incidence of sleeping sickness in northern Ghana during the second World War by simply removing tsetse habitat [5]. In the 1970s–1980s, scientists in West [11] and southern Africa [12] provided the basis for effective tsetse control using odour baits and impregnated tsetse targets and traps. As there is no record of insecticide resistance evolving in tsetse populations, the use of synthetic pyrethroids poses no risk in terms of the need for alternative insecticides. While traps/targets were effective as part of government funded control schemes, they have been shown to suffer from sustainability problems when left to affected communities to handle, related to the ‘tragedy of the commons’ issues. These common goods problems have been shown to be surmountable by the treatment of cattle with insecticide; costs are dramatically reduced when the area of the animal that is treated with insecticide is restricted, encouraging uptake by individual poor cattle keepers [13]. A public–private partnership (Stamp Out Sleeping Sickness, http://www.stampoutsleepingsickness.com/) set up to prevent the overlap of Gambian and Rhodesian (acute) forms of sleeping sickness in Uganda [14] has shown that restricted application of insecticide provides benefits not only by removing the main animal reservoir of Rhodesian sleeping sickness but also for animal health. Given that the distribution of sleeping sickness is limited to ancient and recognised foci, such privately funded and locally adopted approaches have more relevance to control of this disease than the continent-wide approach to eliminate tsetse from Africa [15].

Control of Gambian sleeping sickness depends on the strength of national health systems to provide routine surveillance, effective diagnosis, and drug availability. The classic and successful targeted approach of the dedicated mobile team, however effective in the past, is no longer seen as a priority when services need to be integrated and polyvalent. If the mobile team is no longer a priority, sleeping sickness will continue to be a lingering problem smouldering in the least accessible, poverty-stricken populations and classically in fragile and post-conflict states [16].

Whilst the optimism of some in the sleeping sickness community applauding the nifurtimox/eflornithine announcement is understandable, it is worth remembering that a recommendation is one thing while implementation at scale, by health services prepared to finance it, is another. Even if the perfect silver bullet emerged and was financed, populations that live far from any functioning health facility are those in real need. For the coming decade, only the tried and tested vertical approaches will work if a sustainable impact on Gambian sleeping sickness—a reduction in incidence—is to be achieved. Research cannot deliver in less than that time scale, and we know the classical approaches—early diagnosis by regular surveillance and treatment—actually work. Although WHO has defined sleeping sickness as a “tool deficient disease,” it can be argued that although the tools available are not ideal, tools of proven efficacy do exist. Now is the time to deploy them at scale.

African trypanosomiasis represents a failure of both science and public health [5]. Two failures of responsibility by these diverse and highly divergent communities is not an enviable legacy when previous generations of committed field workers actually reduced the public health problem to one of almost zero incidence. We hope this provides a context and wake-up call to those who fund research and have an interest in actually making a difference to the thousands suffering and dying from sleeping sickness. Research on the trypanosome is not the same as research on sleeping sickness - the two frequently never meet. Trypanosomes may be attractive biological models for the researcher, but these beautiful creatures offer only a grim reality to those afflicted by an inevitably fatal disease. Today we are able to undertake the most elaborate scientific experimentation on tsetse and trypanosomes, yet we are barely able to manage sleeping sickness during the comfort afforded by the present inter-epidemic period. The huge rise in philanthro-capitalist investments that has been welcome in the past decade now needs to translate into practical solutions for rural peoples to manage this devastating disease [17]. Investments that we have seen in genetics and genomics may reap rewards in years to come, but in the meantime, funds must be provided to sustain effective, if unsexy, control strategies. When the next epidemic comes, and it will in the absence of active surveillance and screening, the tacit knowledge will have been lost and we will have to start all over again. It is time that this reality is moved to the forefront and that we all wake up; we have been caught sleeping. The international health community is regularly challenged to deploy “lessons learnt” through many bitter experiences. We feel empowered as both elder practitioners and students of both tsetse and trypanosomes, with a degree of field experience, to recall the famous words of Pete Seeger so pertinent to sleeping sickness, “when will they ever learn, when will they ever learn?”. Let us abandon the notion that trypanosome and tsetse research is synonymous with a case of sleeping sickness or a health system trying to control it.
